# mGluR5 ablation leads to age-related synaptic plasticity impairments and does not improve Huntington’s disease phenotype

**DOI:** 10.1038/s41598-022-13029-z

**Published:** 2022-05-28

**Authors:** Jessica M. de Souza, Talita H. Ferreira-Vieira, Esther M. A. Maciel, Nathalia C. Silva, Izabella B. Quirino Lima, Juliana G. Doria, Isabella G. Olmo, Fabiola M. Ribeiro

**Affiliations:** Department of Biochemistry and Immunology, Institute of Biological Sciences (ICB), Universidade Federal de Minas Gerais, Ave. Antonio Carlos 6627, Belo Horizonte, MG CEP: 31270-901 Brazil

**Keywords:** Cell death in the nervous system, Cognitive ageing, Diseases of the nervous system, Learning and memory, Molecular neuroscience, Neural ageing, Neurotrophic factors, Synaptic plasticity

## Abstract

Glutamate receptors, including mGluR5, are involved in learning and memory impairments triggered by aging and neurological diseases. However, each condition involves distinct molecular mechanisms. It is still unclear whether the mGluR5 cell signaling pathways involved in normal brain aging differ from those altered due to neurodegenerative disorders. Here, we employed wild type (WT), mGluR5^−/−^, BACHD, which is a mouse model of Huntington’s Disease (HD), and mGluR5^−/−^/BACHD mice, at the ages of 2, 6 and 12 months, to distinguish the mGluR5-dependent cell signaling pathways involved in aging and neurodegenerative diseases. We demonstrated that the memory impairment exhibited by mGluR5^−/−^ mice is accompanied by massive neuronal loss and decreased dendritic spine density in the hippocampus, similarly to BACHD and BACHD/mGluR5^−/−^ mice. Moreover, mGluR5 ablation worsens some of the HD-related alterations. We also show that mGluR5^−/−^ and BACHD/mGluR5^−/−^ mice have decreased levels of PSD95, BDNF, and Arc/Arg3.1, whereas BACHD mice are mostly spared. PSD95 expression was affected exclusively by mGluR5 ablation in the aging context, making it a potential target to treat age-related alterations. Taken together, we reaffirm the relevance of mGluR5 for memory and distinguish the mGluR5 cell signaling pathways involved in normal brain aging from those implicated in HD.

## Introduction

The human lifespan has increased during the last century due to scientific advances in biotechnology and medicine^1^. However, with the population over 65 years of age expanding, managing the health and independence of this population is an ongoing concern. One of the main issues afflicting the elderly population is the decline of cognitive abilities, which ultimately affects their quality of life^2^. Understanding the mechanisms underlying cognitive decline may help to determine effective strategies to mitigate these effects.

Learning and memory impairments are associated with aging and many neurological diseases^3–6^. Although various brain regions can be affected by these processes, the hippocampus is the main brain substrate whose alteration leads to cognitive decline in both scenarios^7,8^. Aging and neurodegenerative disorders can lead to decreased number of synapses, reduced neuronal activity, loss of synaptic connections, and synaptic disfunction in the hippocampus^8–11^. However, each condition involves distinct mechanisms to disturb synaptic plasticity, and distinguishing them could be the key to identify molecular targets to treat age-related memory disorders.

Glutamate and its receptors are crucial for spatial learning and hippocampus-dependent memory processes^12^. The metabotropic glutamate receptor 5 (mGluR5) is a G_αq_-coupled receptor, which is abundant in the hippocampus and cerebral cortex^13^. mGluR5 is highly expressed in neurons, where it can modulate the postsynaptic response^14^. Previous studies have demonstrated the important role of mGluR5 in synaptic plasticity, modulating long-term potentiation (LTP) and long-term depression (LTD) and activating cAMP response element-binding protein (CREB) signaling, which is one of the most important proteins for memory formation^15–17^.

Evidence in the literature suggests that aging mice with impaired memory exhibit reduced levels of mGluR5 at the postsynaptic densities in the hippocampus, which culminates in lower activation of its downstream signaling^12,18^. Moreover, studies from our group have shown that mGluR5 ablation accelerates age-related neurodegeneration and neuroinflammation in the cortex^19^. Interestingly, under neuropathological conditions such as Huntington’s disease (HD), mGluR5 also seems to be important for cognitive function^20^. Studies from our group have shown that mGluR5 activation increases the expression of several genes important for synaptic plasticity, reversing the memory deficits exhibited by a mouse model of HD, the BACHD mice^21–23^. However, others have shown that mGluR5 antagonism could alleviate HD-related alterations^24–26^. Although the relevance of mGluR5 for cognitive mechanisms is well established, it is still unclear whether the mGluR5 cell signaling pathways involved in normal brain aging differ from those altered due to neurodegenerative diseases.

To shed some light on this issue, given the potential role of mGluR5 in synaptic plasticity and cognitive function, in the present study we have employed mGluR5^−/−^ mice at 2, 6, and 12 months of age to perform a series of behavioral, histological, and molecular analyses. As a model of progressive neurodegeneration, we choose BACHD, a transgenic mouse model of HD that expresses mutant huntingtin (htt) containing 97 glutamines in the amino-terminal region^27^. BACHD mice have a robust phenotype and exhibit age-dependent neurological decline^22,27^. In addition, to determine whether mGluR5 ablation could exacerbate the phenotype of a mouse model of neurodegenerative diseases, we employed double mutant mice (mGluR5^−/−^/BACHD). The results indicate that mGluR5 is crucial for normal aging and that mGluR5 ablation can worsen some of the deficits exhibited by BACHD mice.

## Materials and methods

### Material

TRIzol (CAT #15596-018), Nuclease-Free Water (CAT #750023) and Power SYBR® Green PCR Master Mix (CAT # #4367659) were purchased from Thermo Fisher Scientific, Waltham, MA, USA. Vecta stain Elite ABC Kit (Mouse IgG and Rabbit IgG) and Vector SG Peroxidase Substrate Kit were purchased from Vector Laboratories (Burlingame, CA, USA). Entellan® (CAT #107960) was from Merck, Kenilworth, NJ, USA. Mouse anti-neuronal nuclei (NeuN) (Cat# MAB377, RRID: AB_2298772) and rat Horseradish peroxidase-conjugated anti-rabbit IgG secondary antibody (CAT #170-6515) were from Bio-Rad Laboratories, Hercules, CA, USA. FD Rapid GolgiStain™ (CAT #PK401) was purchased from FD NeuroTechnologies, Columbia, MD, USA. Rabbit anti-p-CREB and anti-CREB polyclonal antibody (CAT #sc-52, RRID:AB_2629503) were obtained from Cell Signaling Technology, Danvers, Massachusetts, USA. Rabbit anti-vinculin (CAT #129002) were obtained from Abcam, Cambridge, United Kingdom). ECL Western blotting detection reagents (CAT # #RPN2232) were purchased from GE Healthcare, Chicago, IL, USA. 2-chloro-4-((2,5-dimethyl-1-(4-(trifluoromethoxy) phenyl)-1H-imidazol-yl) ethynyl) pyridine (CTEP) was purchased from Axon Medchem. Rabbit anti-mGluR5 (CAT #AB5675) and all other biochemical reagents were purchased from Sigma–Aldrich, Saint Louis, MO, USA.

### Methods

#### Animals

FVB/NJ (wild-type, RRID: IMSR_JAX:001,800) and FVB/N-Tg (HTT*97Q) IXwy/J (BACHD) transgenic mice^27^ and mGluR5 knockout B6; 129-Grm5tm1Rod/J (mGluR5^−/−^) mice^18^ were purchased from The Jackson Laboratory (Bar Harbor, ME, USA). For the generation of double mutant animals, mGluR5^−/−^ and BACHD mice were crossed, obtaining the F1 parental lineage. Afterwards, F1 mice were crossed to obtain the lineages of interest (F2): WT, mGluR5^−/−^, BACHD and BACHD/mGluR5^−/−^ (double mutant). Only F1 mice were used for crosses and the heterozygous F2 mice were discarded, as described previously^19,28^. This study was conducted using littermate male and female mice at the ages of 2, 6 and 12 months. Only male mice were used for behavioral experiments. For all the other experiments, 50% of the animals were male and 50% female. As no sex-difference was observed, data from male and female mice were combined. Mice were housed in an animal care facility at 23 °C on a 12 h light/12 h dark cycle with food and water provided ad libitum. All mice that were euthanized in this study were first anesthetized with ketamine/xylazine (80/8 mg/kg) i.p. before cervical dislocation. Housing and all methods and experimentations were carried out in compliance with the ARRIVE guidelines^29^ and according to the guidelines of the Brazilian National Council of Control of Animal Experimentation (CONCEA) and approved by the Ethics Committee on Animal Use (CEUA) of Federal University of Minas Gerais, under the protocol number CEUA/UFMG 234/2016.

#### Study design

All the experiments were carried out blind. Experimental groups were assigned by a different person than the experimenter and experimenter was unaware of the animal’s group and genotype during experimentation and statistical analysis. WT, mGluR5^−/−^, BACHD and BACHD/mGluR5^−/−^ (double mutant) were allocated in experimental groups by simple randomization. Predicted sample size was calculated using the formula IC = 2 × SD/n − 2 (IC, confidence interval; SD, standard deviation; n, sample size). A total of 23 WT, 26 mGluR5^−/−^, 22 BACHD and 27 BACHD/mGluR5^−/−^ male mice; as well as 11 WT, 11 mGluR5^−/−^, 16 BACHD and 8 BACHD/mGluR5^−/−^ female mice were used for this study. Three male mice (1 WT, 1 BACHD and 1 mGluR5^−/−^) died before 12 months of age and were thus excluded.

#### Y maze test

WT, mGluR5^−/−^, BACHD and BACHD/mGluR5^−/−^ (double mutant) male mice, at 6 and 12 months of age, were submitted to the Y maze test. This test is based on the natural drive of rodents to explore novel environments and measure the short-term spatial working memory. Testing occurs in a Y-shaped maze with three arms, named A, B, and C at a 120° angle from each other. Each arm has a dimension of 30 cm × 6 cm × 20 cm made of light-gray opaque plastic material. All sessions were performed during the first part of the light cycle. The procedure consists in the introduction of the animal to the end of an arm, allowing it to freely explore the three arms for 8 min. The maze was maintained in the same position during the experiment and the animal behavior was recorded by camera. The apparatus was cleaned with 70% ethanol between each session. Parameters analyzed after the end of the session were the number of arm entries (insertion of the four paws of the animal) and the number of triads (three consecutive entries into different arms), in order to calculate the percentage of correct spontaneous alternation with the following formula^30^:

$$Spontaneous\;alternation \, \% \, = \frac{\# triads}{{Total\;number\; \, of\; \, arm \, \;entries \, {-} \, 2}} \times 100$$

#### Object recognition

WT, mGluR5^−/−^, BACHD and BACHD/mGluR5^−/−^ (double mutant) male mice, at 6 and 12 months of age, were submitted to object recognition test, as described previously^21^. This memory test is based on mice preferential spontaneous exploration of objects placed at a novel location. The apparatus used was an open box made of polyvinyl chloride (PVC) 50 cm × 35 cm × 25 cm surmounted by a video camera and a light. Two identical objects made of glass or plastic were used. Objects weight was such that they could not be displaced by mice. As far as we could ascertain, they had no natural significance for mice and they had never been associated with reinforcement. Initial tests showed that mice did not have any preference for the objects used. The general procedure consisted of three different phases: a familiarization phase, a training phase, and a test phase. On the first day, mice were individually submitted to a single familiarization session of 10 min, during which they were introduced to the empty arena. Twenty hours later, animals were submitted to a single 10-min training session, during which two identical objects (object 1 and object 2) were placed in symmetrical positions from the center of the arena and each object was 15 cm from the side walls. Animals did not display object preference during the training phase. After a 90-min delay, during which mice returned to their home cage, animals were reintroduced into the arena for 10 min (test phase) and exposed to the same objects, but one of the objects was displaced to a novel position. The apparatus and the objects were cleaned with 70% ethanol between each session. All sessions were performed during the first part of the light cycle and mice were acclimated to the room for at least 15 min before the beginning of each session. Exploration was defined as sniffing or touching the object with the nose. Only animals that explored both objects for at least 30 s were included. Exploration time of each object was measured during the first 30 s of total exploration. During the test phase, exploration of the modified object for a time longer than 15 s indicates mouse remembered the old location^31^.

#### Immunohistochemistry

Brains from WT, mGluR5^−/−^, BACHD and BACHD/mGluR5^−/−^ (double mutant) mice at 2, 6 and 12 months of age were dissected out and stored in 4% paraformaldehyde (PFA) in phosphate buffered saline (PBS) (137 mM NaCl, 2.7 mM KCl, 8 mM Na_2_HPO_4_, and 2 mM KH_2_PO_4_) for 72 h, as described previously^19,22^. Prior to sectioning, brains were put into 30% sucrose in PBS overnight at 4 °C. Brains were coronally sectioned in cryostat and 30 μm slices were stored in cryoprotect solution. Coronal slices containing both hippocampi were selected for immunohistochemistry analysis using a peroxidase-based immunostaining protocol to label free-floating sections. In brief, endogenous peroxidase activity was quenched using 0.3% hydrogen peroxide, washed 2 times per 5 min with 1 × PBS, after which the membranes were permeabilized using 1% Triton X-100 for 10 min. Non-specific binding was blocked for 30 min using 1.5% horse serum. Slices were incubated with mouse anti-NeuN (1:100) in 2% normal horse serum (from Vector Elite Kit) and 3% bovine serum albumin (BSA) in PBS overnight at 4 °C. Sections were washed in PBS and then incubated in secondary antibody, biotinylated horse anti-mouse (1:400, Vector Elite ABC kit mouse) for 90 min at 4 °C. Finally, sections were incubated in avidin–biotin enzyme reagent complex (Vector Elite Kit) for 90 min at 4 °C, according to manufacturer's instructions. Immunostaining was visualized using a chromogen (Vector SG substrate). Sections were mounted on slides and visualized using an Axio Imager A2-Carl Zeiss Microscope with a Zeiss 20 × lens and representative 710 μm × 532 μm areas of hippocampus was imaged for analyses. The number of NeuN positive puncta per image was counted by a blinded observer using the cell counter tool from ImageJ (NIH, USA, RRID:nif-0000-30467), as previously described^22^.

#### Immunobloting

Hippocampus from WT, mGluR5^−/−^, BACHD and BACHD/mGluR5^−/−^ (double mutant) mice at 2, 6 and 12 months of age were dissected and lysed in RIPA buffer containing SigmaFast™ Protease Inhibitor Cocktail Tablets. Total cellular protein (80 μg) for each sample was subjected to SDS-PAGE, followed by immunoblotting onto nitrocellulose membranes, as described previously^22^. Membranes were blocked with 5% BSA in wash buffer (150 mM NaCl, 10 mM Tris–HCl, pH 7.0, and 0.05% Tween 20) for 1 h and then incubated with either rabbit anti-phospho CREB (S133) (1:1000) or anti-mGluR5 (1:500) antibodies in wash buffer containing 3% BSA at 4 °C overnight. Membranes were rinsed three times with wash buffer and then incubated with secondary peroxidase-conjugated anti-rabbit IgG antibody diluted 1:5000 in wash buffer containing 3% skim milk for 1 h. Membranes were rinsed three times with wash buffer and incubated with ECL Western blotting detection reagents. Antibodies were then stripped and membranes were incubated with either rabbit anti-CREB (1:1000) or anti-vinculin (1:1000) antibodies for 2 h and probed with secondary antibody anti-rabbit IgG diluted 1:5000 to determine total CREB expression. Non-saturated, immunoreactive CREB and mGluR5 bands were quantified by scanning densitometry. Immunoband intensity was calculated using Image J™ software to determine the number of pixels of CREB bands.

#### Quantitative RT-PCR

Hippocampus from WT, mGluR5^−/−^, BACHD and BACHD/mGluR5^−/−^ (double mutant) mice at 2, 6 and 12 months of age were dissected and used for RNA extraction, as described previously^32^. RNA was isolated using TRIzol® reagent as per manufacturer’s instructions. RNA was re-suspended in 20 μL of nuclease-free water, and its concentration and quality were analyzed by NanoDropTM (Thermo Scientific) and gel electrophoresis, respectively. cDNAs were prepared from 2 μg of total RNA extracted in a 20 μL final reverse transcription reaction. Quantitative RT-PCR (RT-qPCR) was performed using the Power SYBR® Green PCR Master Mix in the QuantStudio™ 7 Flex real-time PCR system platform (Applied Biosystems). RT-qPCR was performed to quantify mRNA levels of the brain-derived neurotrophic factor—BDNF (NM_001285416.1), activity regulated cytoskeleton associated protein—Arc/Arg3.1 (NM_018790.3), syntaxin 1A (NM_016801.3), and post-synaptic density protein (PSD95) – Dgl4 (NM_007864.3). Primers were designed using Primer3Plus Program^33^. BDNF (forward: 5′ ATGAAAGAAGTAAACGTCCAC 3′; reverse 5′ CCAGCAGAAAGAGTAGAGGAG 3′), Arc/Arg3.1 (forward 5′ GCTGAAGCAGCAGACCTGA 3′; reserve 5′ TTCACTGGTATGAATCACTGCTG 3′), syntaxin 1A (forward: 5′ GAACAAAGTTCGCTCCAAGC 3′; reverse: 5′ GTGGCGTTGTACTCGGACAT 3′) and PSD-95 (forward: 5′ TCTGTGCGAGAGGTAGCAGA 3′; reverse 5′ AAGCACTCCGTGAACTCCTG 3′). The BDNF set of primers allows the detection of all the 12 BDNF transcript variants. Previous verification of undesired secondary formations or dimers between primers were performed using ‘OligoAnalyser 3.1’ tool (Integrated DNA Tecnology©), available at https://www.idtdna.com/calc/an analyzer. All primers used in this work were validated by serial dilution assay and the reaction efficiency was calculated, comprising 90–110% (data not shown). Samples were prepared in triplicate and changes in gene expression were determined with the 2^−ΔCt^ method using actin for normalization.

#### Golgi-Cox staining

Male WT, mGluR5^−/−^, BACHD and BACHD/mGluR5^−/−^ (double mutant) mice at 2, 6 and 12 months of age were killed and brains were removed and stained using the FD Rapid GolgiStain™ kit (FD NeuroTechnologies), following manufacturer’s instructions and as described previously^21^. Briefly, brains were immersed in an impregnation solution (A + B), which was replaced after 6 h, and then kept in dark for 14 days. Afterward, brains were transferred into solution C, which was replaced after 24 h, and kept in dark for 72 h. Brains were coronally sectioned generating 100 μm slices using a cryostat. Slices were then mounted on gelatin-coated microscope slides, stained, dehydrated, and cover-slipped with Entellan®.

#### Image acquisition and dendritic spine analyses

Pyramidal hippocampal neurons were chosen for analyses according to the following criteria: (i) Cell completely filled with Golgi-Cox stain; (ii) No overlap with other cells to ensure no bias of confusion; (iii) Cell body well defined; (iv) Intact primary dendrite ramification; (v) Presence of secondary or tertiary ramification measuring at least 20 μm. A total of 12–15 Z-stack images per animal were acquired using a confocal microscope (Nikon Eclipse C2 with a × 63 objective-oil) by a blinded observer. Measurement and classification of the dendritic spines were performed using the free software Image J™ and RECONSTRUCT, available at (http://synapses.clm.utexas.edu), as previously described^34^.

#### Data analyses

Means ± SEM are shown for the number of mice indicated in each graph. GraphPad Prism™ software was used to analyze data for statistical significance and for curve fitting. Data were tested for normality of distribution by the D’Agostino&Pearson omnibus normality test. No outliers (greater than two deviations from the mean) were found and, thus, no data was excluded. Statistical significance (p < 0.05) was determined by unpaired Student t-test, one-way or two-way analysis of variance (ANOVA), followed by Bonferroni or Sidewalk post hoc multiple comparison testing, as stated in the figure legends.

## Results

### mGluR5 ablation promotes hippocampal neuronal loss in aged mice

Many studies have proposed that the loss of neurons in the hippocampus is a morphological correlate of the memory impairment seen during aging or due to the progression of dementia^35^. It is well-known that mGluR5 plays an important role in neuronal survival/neurodegeneration^19,36,37^. However, the role of mGluR5 in hippocampal neuronal maintenance during aging is unknown. Thus, we decided to evaluate whether mGluR5 knockout mice could display different levels of neuronal loss in an age-dependent manner. Therefore, we performed immunohistochemistry experiments using an antibody to label the neuronal specific marker, NeuN, to assess neuronal cell loss in hippocampal slices from mGluR5^−/−^ and control mice (WT) at 2, 6 and 12 months of age. To compare the role of mGluR5 in normal aging and in a neurodegenerative process that progresses with aging, we employed the BACHD mice, which is a mouse model of HD that has a robust and progressive neurodegenerative phenotype^27^, as well as double mutant mice (BACHD/ mGluR5^−/−^), which express mutant huntingtin protein in the absence of mGluR5 expression.

**Figure 1:**
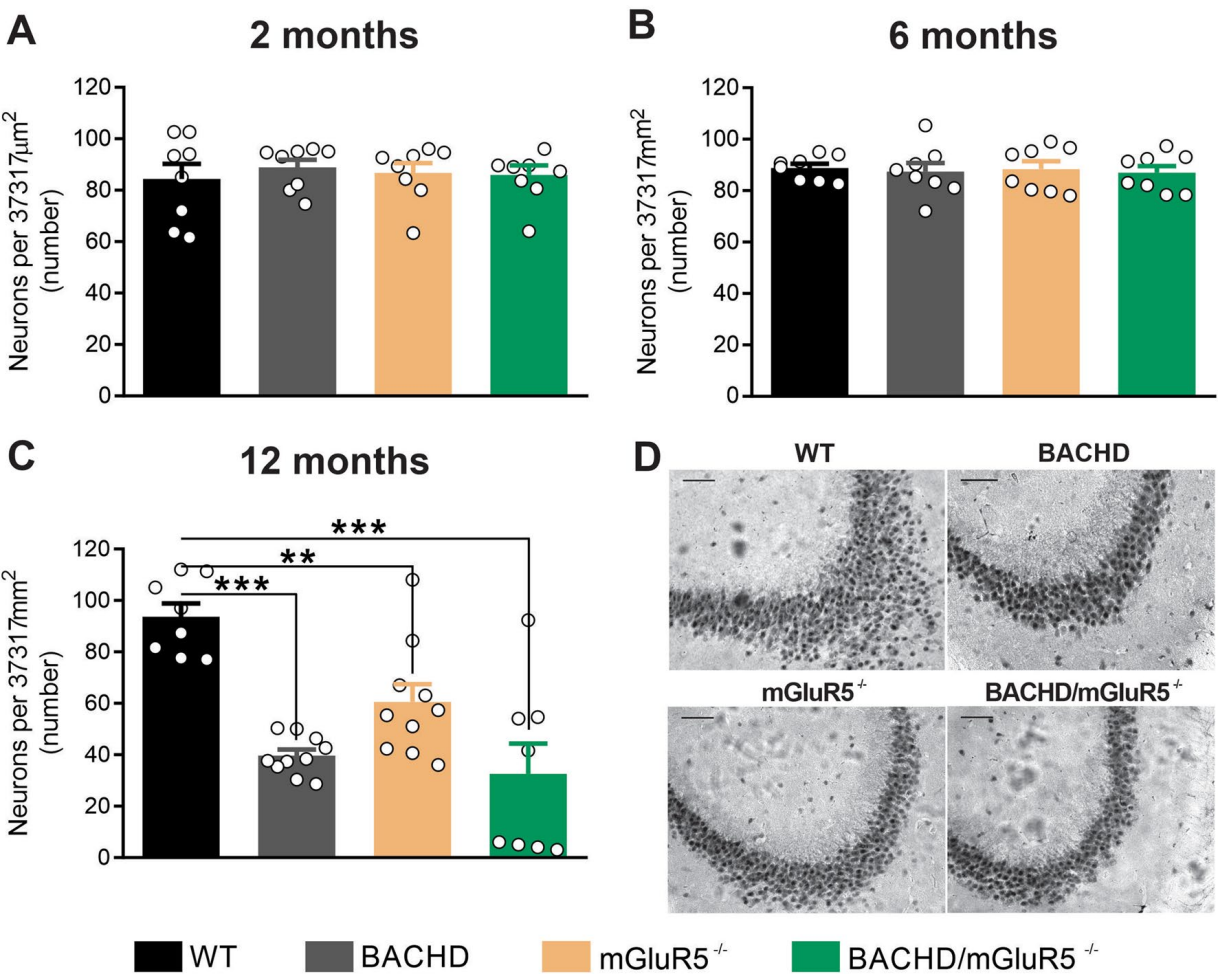
mGluR5 ablation promotes hippocampal neuronal loss in aged mice. Graphs show quantification of NeuN-labeled hippocampal neurons per 37,317 μm^2^ in wild-type (WT), BACHD, mGluR5^−/−^ and BACHD/mGluR5^−/−^ mice at 2 (n = 8) (**A**), 6 (n = 8) (**B**) and 12 (n = 8–10) (**C**) months of age. (**D**) Shown are representative images for NeuN immunostaining of hippocampal slices from 12-month-old WT, BACHD, mGluR5^−/−^ and BACHD/mGluR5^−/−^ mice. Scale bar = 100 μm. Data represent the means ± SEM obtained from 6 images taken from 2 histological slices per mouse. **p < 0.01 and ***p < 0.001 by one-way ANOVA and Bonferroni's multiple comparisons test.

At 2 and 6 months of age, there was no difference in the number of hippocampal neurons among groups (Fig. 1A,B). However, 12-month-old BACHD, mGluR5^−/−^ and BACHD/mGluR5^−/−^ mice exhibited a reduction in the number of neurons in the hippocampus, as compared to WT mice (Fig. 1C,D). Interestingly, no additive effect was observed between mutant huntingtin (mHTT) expression and mGluR5 ablation regarding neurodegeneration, as BACHD phenotype was not altered due to mGluR5 ablation. These data indicate that mGluR5 ablation, similarly to mHTT, promotes age-related neurodegeneration in the hippocampus.

### Lack of mGluR5 decreases dendritic spine density and maturation in the hippocampus of aged mice

Dendritic spines are neuronal protrusions that mainly mediate excitatory synaptic transmission in the mammalian brain and are essential for learning and memory^38,39^. Normal aging and neurodegeneration are accompanied by dendritic regression and spine loss, contributing to cognitive decline^40^. Since mGluR5 has been implicated in dendrogenesis and spineogenesis^41^, we investigated whether mGluR5 ablation in aging mice would lead to morphological alterations of postsynaptic terminals and changes in the number of synaptic terminals.

**Figure 2:**
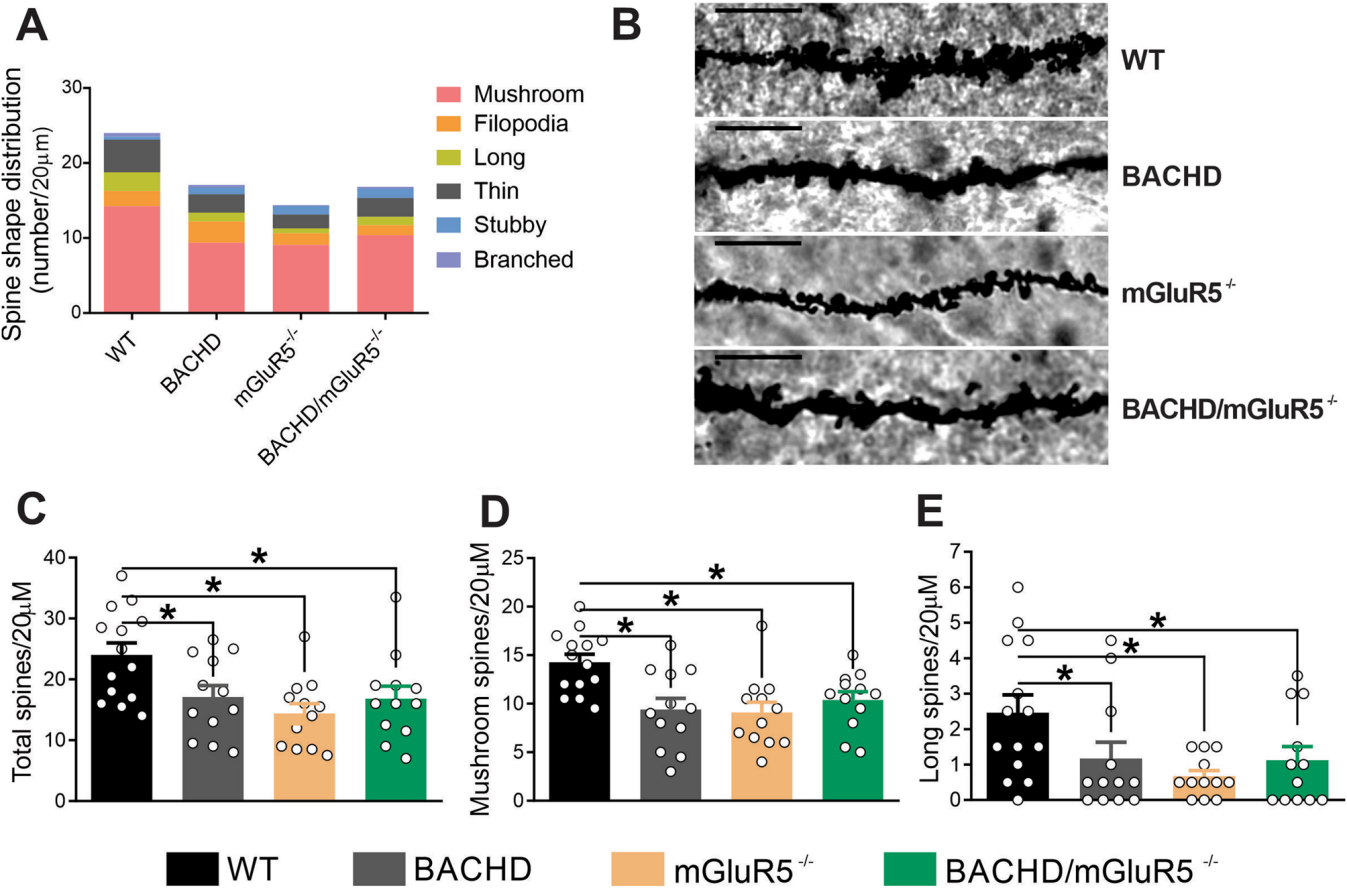
Lack of mGluR5 decreases dendritic spine density and maturation in the hippocampus of aged mice. (**A**) Colored graph shows the contribution of each type of spine per 20 µm of dendrite segment in the hippocampus of wild-type (WT), BACHD, mGluR5^−/−^ and BACHD/mGluR5^−/−^ mice at 12 months of age (n = 10–12). (**B**) Shown are representative confocal micrographs from tertiary dendrites of hippocampal neurons from wild-type (WT), BACHD, mGluR5^−/−^ and BACHD/mGluR5^−/−^ mice at 12 months of age. Dendrite segments shown are 20 µm in length and size bar corresponds to 5 µm. Graphs show total number of dendritic spines (**C**) and the number of mature mushroom (**D**) and transient long (**E**) spines per 20 µm of dendrite segment. Data represent the means ± SEM. *p < 0.05 by one-way ANOVA and Bonferroni's multiple comparisons test.

For that, we employed the Golgi–Cox impregnation technique to analyze dendritic spines in the hippocampus of WT, BACHD, mGluR5^−/−^ and BACHD/mGluR5^−/−^ mice at 12 months of age. The overall morphology and number of dendritic spines highlighted a different pattern in BACHD, mGluR5^−/−^ and BACHD/mGluR5^−/−^, as compared to WT mice (Fig. 2A,B). Statistical analyses showed that BACHD, mGluR5^−/−^ and BACHD/mGluR5^−/−^ mice exhibited decreased number of dendritic spines, as compared to WT animals (Fig. 2B,C). Regarding dendritic spine morphology, BACHD, mGluR5^−/−^ and BACHD/mGluR5^−/−^ displayed a reduction in the number of both transient and mature spines, represented by long and mushroom-like spines, respectively (Fig. 2D,E). These results indicate that mGluR5 ablation is as detrimental to spine establishment and maturation as mutant HTT expression in the aged hippocampus. However, mGluR5 ablation did not induce further spine loss in BACHD mice, as the number of dendritic spines were not different when comparing BACHD and double mutant mice.

### Lack of mGluR5 leads to cognitive impairment

Aging is associated with a decline in hippocampal-dependent memory^42^. It is well known that mGluR5 signaling is required for memory formation and consolidation. As we observed neuronal loss and reduced dendritic spine number in aging mice, we decided to investigate whether mGluR5 ablation would lead to cognitive deficits.

**Figure 3:**
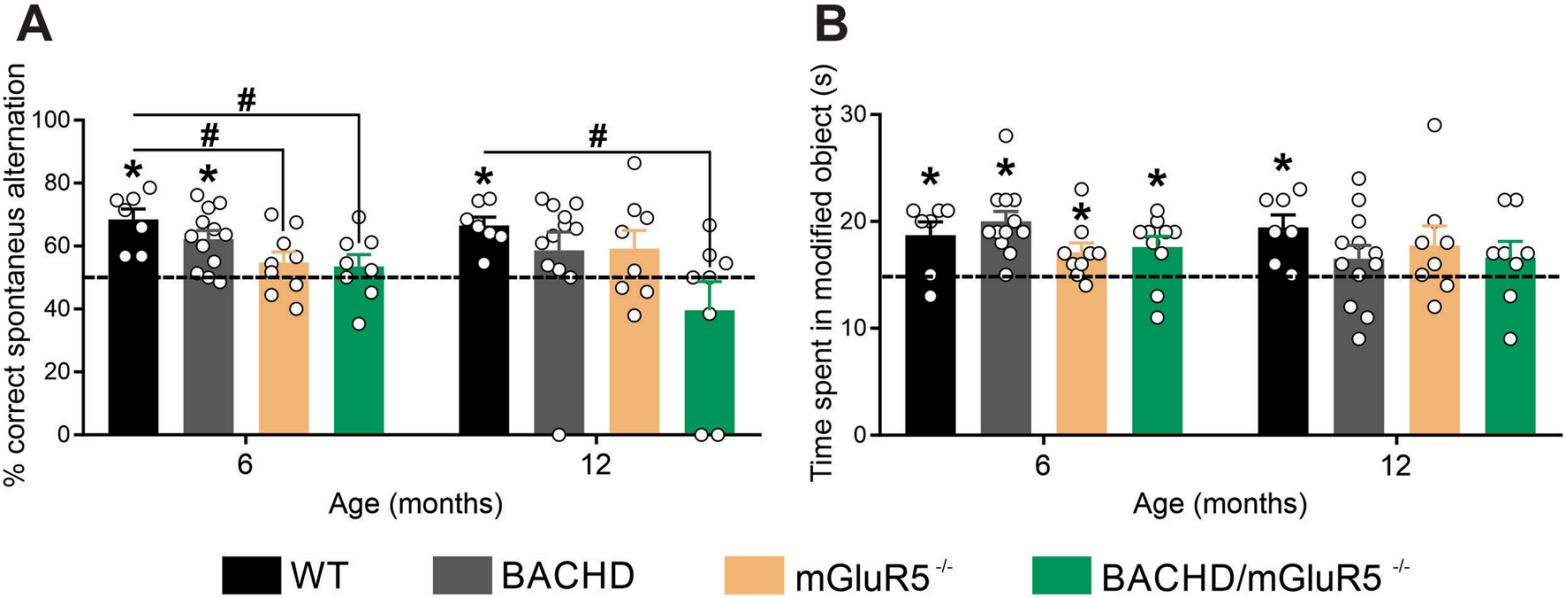
Lack of mGluR5 leads to cognitive impairment. (**A**) Graph shows percentage (%) of spontaneous alternation by wild-type (WT), BACHD, mGluR5^−/−^ and BACHD/mGluR5^−/−^ mice at 6 (n = 7–12) and 12 (n = 6–12) months of age, calculated as the correct spontaneous changes considering the total number of arm entries. A score superior to 50% indicates intact working memory. Data represent the means ± SEM. *Indicates significant correct alternation index in relation to chance (50%), p < 0.05 by unpaired Student t-test. ^#^Indicates significant difference as compared to WT, p < 0.05 by two-way ANOVA and Sidewalk's multiple comparisons test. (**B**) Graph shows the time WT, BACHD, mGluR5^−/−^ and BACHD/mGluR5^−/−^ mice, at 6 (n = 7–12) and 12 (n = 7–12) months of age, explored the object placed at the novel location during the first 30 s (s) of total exploration. Values higher than 15 s indicates mouse remembered the old location. Data represent the means ± SEM. *Indicates significant difference in relation to chance (15 s), *p* < 0.05 by unpaired Student t-test.

For that, WT, BACHD, mGluR5^−/−^ and BACHD/mGluR5^−/−^ mice, at 6, and 12 months of age, were submitted to the Y maze test, which evaluates working memory based on animals' natural curiosity for exploration. The total number of entries in the arms was similar among the tested animals (data not shown). WT animals exhibited correct spontaneous alternation higher than chance (50%) at all tested ages (Fig. 3A), indicating that these mice have intact working memory. However, BACHD mice exhibited a poor performance at the Y maze test, showing correct spontaneous alternation higher than chance only at the age of 6 months (Fig. 3A). Moreover, to our surprise, both mGluR5^−/−^ and BACHD/mGluR5^−/−^ exhibited compromised working memory, as these animals did not exhibit correct spontaneous alternation higher than chance at 6 and 12 months of age. In fact, mGluR5^−/−^ animals exhibited a worse performance than that of WT animals at 6 months of age, while double mutant mice performed worse than WT animals at 6 and 12 months of age. These data suggest that mGluR5 ablation is more detrimental to working memory than mHTT expression and that mGluR5 knockout contributes to working memory deficits in both normal aging and diseased mice.

We also analyzed spatial memory deficits through object recognition test, which is based on mouse preference for objects placed at a new location. WT animals, at 6 and 12 months of age, spent more time exploring the object placed at the novel location than the object that remained at the familiar location (Fig. 3B), which indicates that these animals have intact spatial memory. However, BACHD, mGluR5^−/−^ and BACHD/mGluR5^−/−^ mice exhibited impaired memory later in life, as 12-month-old animals were unable to discriminate between objects placed at the familiar and novel location (Fig. 3B). No additive effect was observed between mGluR5 ablation and mHTT expression, indicating that mGluR5 knockout does not induce further memory deficits in BACHD mice. Taken together, these data indicate that mGluR5 knockout triggers memory alterations in both WT and BACHD mice. Of note, mGluR5 ablation seems to be more harmful to memory than mHTT during aging processes.

### Lack of mGluR5 leads to decreased CREB signaling in the aged hippocampus

**Figure 4:**
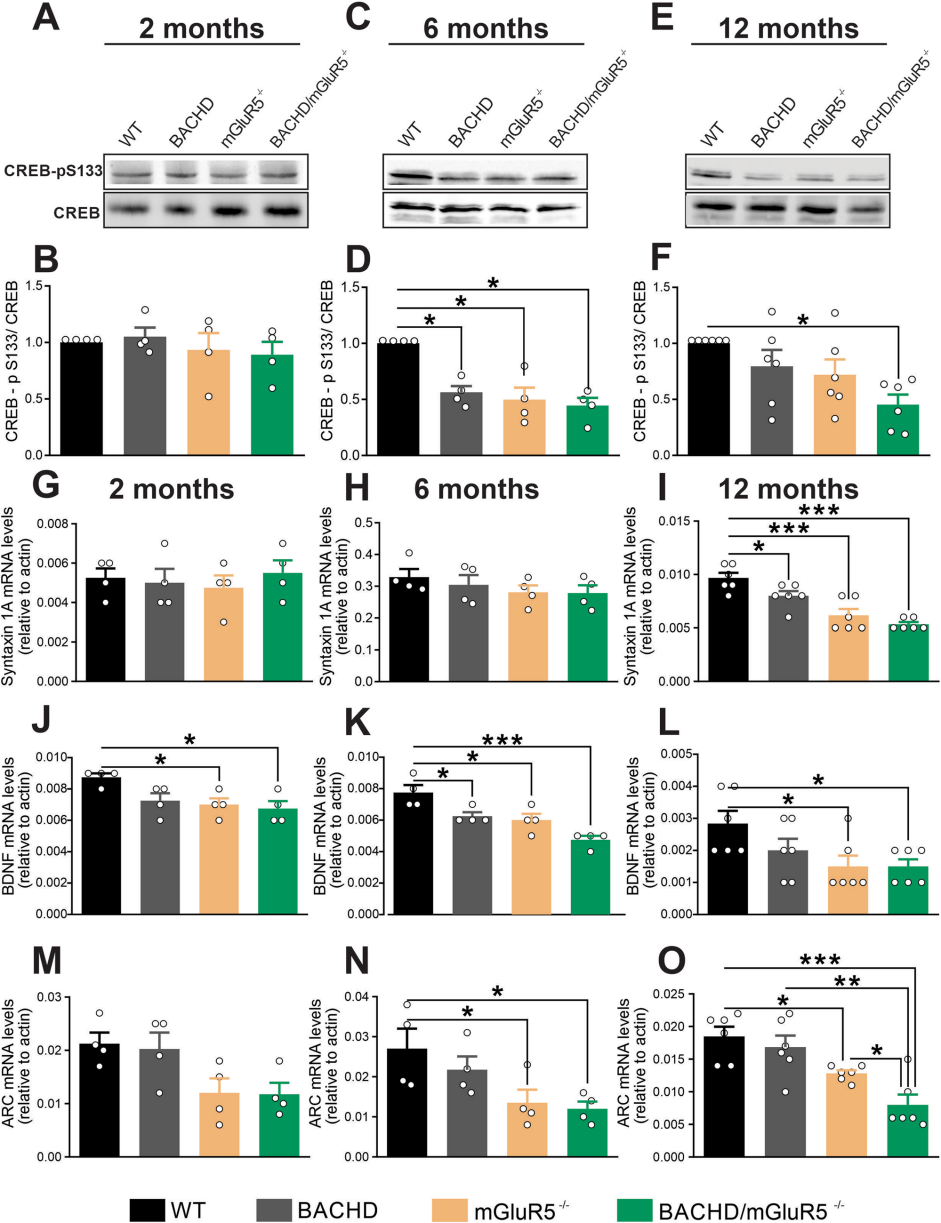
Lack of mGluR5 decrease CREB activation and reduce Syntaxin, BDNF, and Arc/Arg3.1 expression in the hippocampus. Shown are representative immunoblots for CREB-p (S133) (upper panel) and total-CREB (lower panel) expression in the hippocampus of wild-type (WT), BACHD, mGluR5^−/−^ and BACHD/mGluR5^−/−^ mice at 2 (**A**), 6 (**C**) and 12 (**E**) months of age. 80 µg of total cell lysate was used for each sample. Graphs show the densitometric analysis of phosphor-CREB normalized to total-CREB expression in the hippocampus of WT, BACHD, mGluR5^−/−^ and BACHD/mGluR5^−/−^ mice at 2 (n = 4) (**B**), 6 (n = 4) (**D**) and 12 (n = 6) (**F**) months age. Graphs show Syntaxin 1A (**G**, **H** and **I**), BDNF (**J**, **K** and **L**), and Arc/Arg3.1 (**M**, **N** and **O**), mRNA levels in the hippocampus of wild-type (WT), BACHD, mGluR5^−/−^ and BACHD/mGluR5^−/−^ mice at 2 (n = 4) (G and J), 6 (n = 4) (**H** and **K**) and 12 (n = 6) (**I** and **L**) months of age. mRNA levels were assessed by RT-qPCR, which was performed in triplicate and normalized to actin mRNA levels. Data represent the means ± SEM. *p < 0.05 and ***p < 0.01 by one-way ANOVA and Bonferroni's multiple comparisons test.

Aging is associated with specific alterations in signal transduction pathways, which culminates in memory impairments^43^. To detect the molecular and cellular mechanisms underlying this physiological event is a big challenge. It is well-known that mGluR5 initiates a wide variety of cell signaling pathways important for synaptic plasticity^44,45^, including the activation of cAMP response element-binding protein (CREB), a transcription factor that is considered a universal modulator of memory formation^46^. Therefore, we tested whether mGluR5 ablation could change CREB phosphorylation in the aged hippocampus. At 2 months of age, there was no difference in CREB phosphorylation among groups (Fig. 4A,B). At 6 months of age, CREB phosphorylation was reduced in BACHD, mGluR5^−/−^ and BACHD/mGluR5^−/−^ mice, as compared to WT mice (Fig. 4C,D). At 12 months of age, only double mutant mice showed a significant reduction in CREB phosphorylation (Fig. 4E,F), suggesting that mGluR5 further decreases CREB activation in BACHD mice. Moreover, these data indicate that mGluR5 ablation, similarly to mHTT expression, promotes a reduction in CREB phosphorylation in the hippocampus. It is important to note that mGluR5 expression in the hippocampus was not altered by age or the presence of mHTT in WT and BACHD mice (Supplementary Fig. 1A,B). Moreover, mGluR5 ablation was confirmed in mGluR5^−/−^ and BACHD/mGluR5^−/−^ mice (Supplementary Fig. 1A,B).

Since mGluR5 ablation decreased CREB signaling in both WT and BACHD mice, we decided to investigate the expression of syntaxin 1A, which is positively regulated by CREB and is widely used as a presynaptic marker^47^. At 2 and 6 months of age, there was no difference in syntaxin 1A mRNA levels among groups (Fig. 4G,H). However, 12-month-old BACHD, mGluR5^−/−^ and BACHD/mGluR5^−/−^ mice exhibited a reduction in syntaxin 1A mRNA levels, as compared to WT mice (Fig. 4I). These results show an aging-related disturbance in syntaxin 1A expression following both mGluR5 knockout and mHTT expression.

Brain-derived neurotrophic factor (BDNF) plays a fundamental role during age-related synaptic loss, preventing cerebral atrophy and cognitive decline^48^. Interestingly, BDNF is also a downstream target of CREB signaling^49^ and its expression can be mediated by mGluR5^22,50^. To test the effect of mGluR5 ablation in BDNF expression in aging mice, RT-qPCR was performed to measure BDNF mRNA levels in the hippocampus of WT, BACHD, mGluR5^−/−^ and BACHD/mGluR5^−/−^ mice at 2, 6, and 12 months of age. mGluR5^−/−^ and BACHD/mGluR5^−/−^ mice showed decreased BDNF mRNA levels at all analyzed ages (Fig. 4J–L), whereas BACHD mice showed reduced BDNF mRNA levels only at 6 months of age (Fig. 4J–L), indicating that mGluR5 knockout has a more striking effect on BDNF expression than mHTT and that mGluR5 knockout decreases the expression of BDNF in both control and diseased mice.

It has been demonstrated that BDNF can increase the expression of the activity-regulated and cytoskeletal-associated (Arc/Arg3.1) protein^51^, which is essential for the consolidation of synaptic plasticity, contributing to cognition^52,53^. We have previously shown that mGluR5 activation leads to an increase in Arc/Arg3.1 expression^21^. As Arc/Arg3.1 expression is a downstream target of CREB and its expression has not yet been analyzed under mGluR5 ablation, we assessed Arc/Arg3.1 mRNA levels in the hippocampus. At 2 months of age, there was no difference in Arc/Arg3.1 mRNA levels among groups (Fig. 4M). At 6 and 12 months of age, mGluR5^−/−^ and BACHD/mGluR5^−/−^, but not BACHD mice, exhibited a reduction in Arc/Arg3.1 mRNA levels, as compared to WT animals (Fig. 4N,O). Interestingly, at 12 months of age, mGluR5 ablation and mHTT expression had an additive effect regarding Arc/Arg3.1 expression, as double mutant mice exhibited decreased Arc/Arg3.1 mRNA levels as compared to mGluR5^−/−^ and BACHD mice (Fig. 4O). Thus, mGluR5 ablation decreases Arc/Arg3.1 expression in both normal aging (WT) and diseased mice (BACHD). Taken together, these results show that the lack of mGluR5 evokes an early impairment in synaptic plasticity at the molecular level in the hippocampus, which is sustained in aged mice.

### Lack of mGluR5 decreases PSD95 expression in aged mice

**Figure 5:**
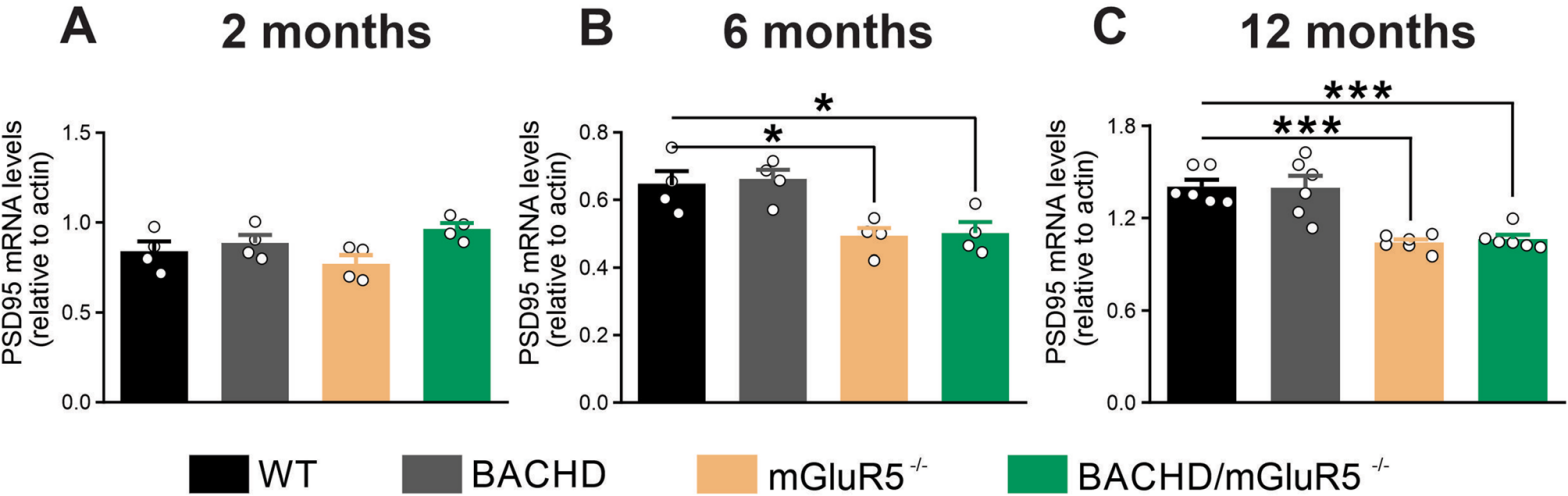
Lack of mGluR5 decreases PSD95 expression in aged mice. Graphs show PSD95 (**A**, **B** and **C**) mRNA levels in the hippocampus of wild-type (WT), BACHD, mGluR5^−/−^ and BACHD/mGluR5^−/−^ mice at 2 (n = 4) (**A**), 6 (n = 4) (**B**) and 12 (n = 6) (**C**) months of age. mRNA levels were assessed by RT-qPCR, which was performed in triplicate and normalized to actin mRNA levels. Data represent the means ± SEM. *p < 0.05 and ***p < 0.01 by one-way ANOVA and Bonferroni's multiple comparisons test.

Alteration in postsynaptic density (PSD) protein expression and a reduction in PSD area have been associated with memory impairments in aged Long-Evans rats^54^. Moreover, mGluR5 is an important modulator of postsynaptic responses at the perisynapse. To verify the consequences of mGluR5 ablation for the postsynaptic region, we analyzed the mRNA levels of PSD95 in the hippocampus. At 2 months of age, there was no difference in PSD95 mRNA levels among groups (Fig. 5A). However, at 6 and 12 months of age, mGluR5^−/−^ and BACHD/mGluR5^−/−^ mice exhibited a reduction in PSD95 mRNA levels, as compared to WT mice (Fig. 5B,C), indicating that mGluR5 affects PSD95 expression in normal aging (WT), as well as in diseased animals (BACHD). Interestingly, these results showed that only mGluR5 ablation, but not mHTT expression, influenced PSD95 expression. We also found similar results regarding the levels of PSD95 mRNA in the striatum and cerebral cortex of these animals (data not shown). Thus, these results indicate that PDS95 may be a specific mGluR5 molecular target in the aging context.

## Discussion

The effects of aging in the brain are numerous and are just beginning to be understood. Advancing of age alters the biological, chemical, and physical functions of neurons, leading to memory impairment, altered behaviors, loss of cognitive functions, dementia, and impaired immune responses^55^. Since mGluR5 displays an unquestionable role in synaptic plasticity and brain physiology^13,56^, various studies have considered this receptor as a therapeutic target for the treatment of cognitive deficits present in normal aging, as well as in neurodegenerative disorders^17,19,36,57,58^. In the present study, we demonstrated that the memory impairment exhibited by mGluR5^−/−^ mice at 12 months of age is accompanied by massive neuronal cell loss and decreased dendritic spine density and maturation in the hippocampus, similarly to BACHD and BACHD/mGluR5^−/−^ mice. Moreover, the results show that the knockout of mGluR5 does not improve the HD-related phenotype and that ablation of the receptor worsens some of the HD-related alterations. Therefore, we propose that mGluR5 is crucial to normal aging and neuronal survival.

Our results confirm well-documented findings showing that both mGluR5 ablation and mHTT expression lead to CREB signaling decline^21,59–63^. It is tempting to view neurodegenerative diseases as expressions of accelerated aging^64^, especially when aging and neurodegenerative insults culminate in similar impairments for the brain. However, this simplification is unhelpful because it does not accurately capture the underlying mechanisms that dissociate normal from pathological aging. Our results demonstrate that mGluR5 knockout has a more striking effect than mHTT on the expression of genes important for synaptic plasticity and memory as mice age. A previous study from our group has shown that ablation of mGluR5 provokes more intense aging-related neuroinflammation than the expression of mHTT^19^. Moreover, it is important to note that the disturbance in working memory was more noticeable in mGluR5^−/−^ and BACHD/mGluR5^−/−^ animals, as compared to BACHD mice. Thus, mGluR5 stimulation could be a positive factor for healthy aging. However, one of the limitations of this study was that only male mice were employed for the behavior tests. Future studies will be important to demonstrate whether the mGluR5-dependent aging effect on memory observed in male mice could be reproduced in female animals.

Ablation of mGluR5 led to cognitive impairment at 6 months of age, although neuronal cell loss in the hippocampus could only be detected in 12-month-old mice. Thus, neuronal cell loss could not be the main factor accounting for the memory deficits. We hypothesize that the age-related memory disturbance caused by mGluR5 ablation could be a consequence of specific changes in cell signaling pathways important to synaptic plasticity. Ablation of mGluR5 led to decreased levels of PSD95, BDNF and Arc/Arg3.1 at 6 and 12 months of age. These proteins are highly relevant to memory and synaptic plasticity in the hippocampus^65–68^. Previous studies have shown that Arc/Arg3.1 knockout mice fail to form long-lasting memories required for implicit and explicit learning tasks and exhibit alterations in long-term depression (LTP)^68^. Moreover, PSD-95 deficiency disrupts synaptic function and related behavior^69^. Changes in BDNF expression are associated with memory loss in normal and pathological aging and in psychiatric disease^70^. Together, these results demonstrate a critical role of mGluR5 as a modulator of the expression of essential proteins involved in the consolidation of synaptic plasticity and memory storage. Genome-wide gene expression studies focusing on aging have revealed age-dependent changes in the expression of a few broadly conserved functional categories of genes^71^. These studies provide evidence of reduced mitochondrial and synaptic function during aging, which is a common feature of a variety of neurodegenerative disorders, such as HD and AD^72–74^. However, it is unknown what are the trigger mechanisms behind these findings in each condition. Alterations in BDNF and Arc/Arg3.1 expression are a common feature between aging and HD^21,75–77^. However, our data indicate that mGluR5 ablation has a more striking effect than mHTT on the expression of genes important for synaptic plasticity in aged mice, as we observed a decrease in the expression of Arc/Arg3.1 and PSD95 only when mGluR5 was absent. Regarding BDNF expression, the effect of mGluR5 ablation was also more pronounced than that of mHTT expression, as we observed decreased BDNF expression in both mGluR5^−/−^ and double mutant mice at 6 and 12 months of age, whereas BACHD mice displayed only a mild decrease in BDNF at 6 months of age and no significant alteration at 12 months of age. Thus, we hypothesize that this striking effect of mGluR5 ablation in the expression profile of PSD95, BDNF, and Arc/Arg3.1 is the main factor contributing to the memory deficits exhibited by aging mGluR5^−/−^ and double-mutant mice. These data also highlight that the mGluR5-dependent molecular mechanisms underlying normal aging might be different than those triggering dementia. However, although the data shown here indicate that there is a temporal correlation between changes in the expression of genes important for synaptic plasticity and mGluR5-dependent memory deficits, future studies will be important to demonstrate whether normalizing the expression of these genes could rescue memory alterations.

Our results demonstrate that in aged mice mGluR5 ablation is as detrimental as mHTT expression and that the knockout of mGluR5 worsens the memory deficits and decreases the activation of CREB and the expression of synaptic genes in BACHD mice. However, most of our results do not show a cumulative effect in double mutant mice, as compared to each single mutant, BACHD or mGluR5^−/−^. Interestingly, at 12 months of age, mGluR5 ablation and mHTT expression had an additive effect only in the case of Arc/Arg3.1 expression. Although an additive outcome was expected, these results could be explained by the fact that these two insults act on the same cell signaling pathways. Corroborating this hypothesis, CREB signaling is a molecular pathway modulated by both mGluR5 and mHTT. mGluR5 stimulation leads to adenylyl cyclase 1 (AC1), calcium/calmodulin-dependent protein kinase IV (CaMKIV), and extracellular signal-regulated protein kinases 1 and 2 (ERK1*/*2) activation, which are proteins well known for triggering CREB phosphorylation and inducing the transcription of genes crucial for synaptic plasticity, such as BDNF^44^. On the other hand, mHTT aggregates can sequester CREB-binding protein (CBP), an important activator of CREB, which suppresses the expression of CREB target genes^78^. In line with these reports, our results show a reduction in CREB phosphorylation in BACHD, mGluR5^−/−^ and BACHD/mGluR5^−/−^, which was followed by a decrease of BDNF and syntaxin 1A expression. However, it is important to note that in aged double-mutant mice the quantitative pattern of expression of BDNF and syntaxin 1A is very similar to the mGluR5^−/−^ mice, but not to BACHD mice. Thus, the crosstalk between mGluR5 and mHTT leads to the modulation of the same cell signaling pathways, but mGluR5 ablation seems to have a preponderant effect than that of mHTT.

Efforts to develop evidence-based treatment strategies for aging are ongoing, but neither highly effective treatments nor potent protective approaches have yet been identified^79^. In the present study, our results show that mGluR5, but not mHTT, regulates PSD95 transcription as only mGluR5^−/−^ and BACHD/mGluR5^−/−^ mice showed a reduction in PSD95 mRNA levels. Previous studies have shown that PSD95 levels are decreased in R6/1 and R6/2 mice, which are transgenic HD mouse models expressing only the amino-terminal region of the mHTT protein^80–82^. However, these results were not replicated in other mouse models that more closely recapitulate HD^83^, which is in agreement with the data shown here indicating that PSD95 levels are not changed by mHTT. In this study we show that mGluR5 affects PSD95 expression in normal aging (WT), as well as in diseased animals (BACHD), which highlights the importance of mGluR5 in aging events independently of other insults. The presence and size of PSD95 clustering are closely associated with dendritic spine growth and structural maintenance, which are important for learning and memory^84^. Interestingly, studies using a mouse model of aging, the SAMP (senescent accelerated prone) mice, show that aging affects PSD-95 expression^85^. The mechanisms involved in this reduction is still unclear. Moreover, PSD95 content was reduced in the hippocampal formation of aged-learning impaired compared to aged-unimpaired and young rats^86^. Thus, PDS95 may be an mGluR5-especific molecular target in the aging context, and its modulation may be helpful to improve cognitive deficits present in the aging population. Taken together, we reaffirm the relevance of mGluR5 for memory and synaptic plasticity and distinguish the mGluR5 cell signaling pathways involved in normal brain aging from those implicated in HD.

## Supplementary Information

Supplementary Figures.

## Data Availability

The datasets generated during and/or analyzed during the current study are available upon reasonable request and will be provided by the corresponding author F.M.R. (fmribeiro@icb.ufmg.br).
