# Supplementary material for: mGluR5 ablation leads to age-related synaptic plasticity impairments and does not improve Huntington’s disease phenotype
Source: Sci Rep. 2022 May 28;12:8982. doi: 10.1038/s41598-022-13029-z (PMC9148310; doi:10.1038/s41598-022-13029-z)
Supplement: Supplementary file 1 — Supplementary Figures. [file 41598_2022_13029_MOESM1_ESM.docx]

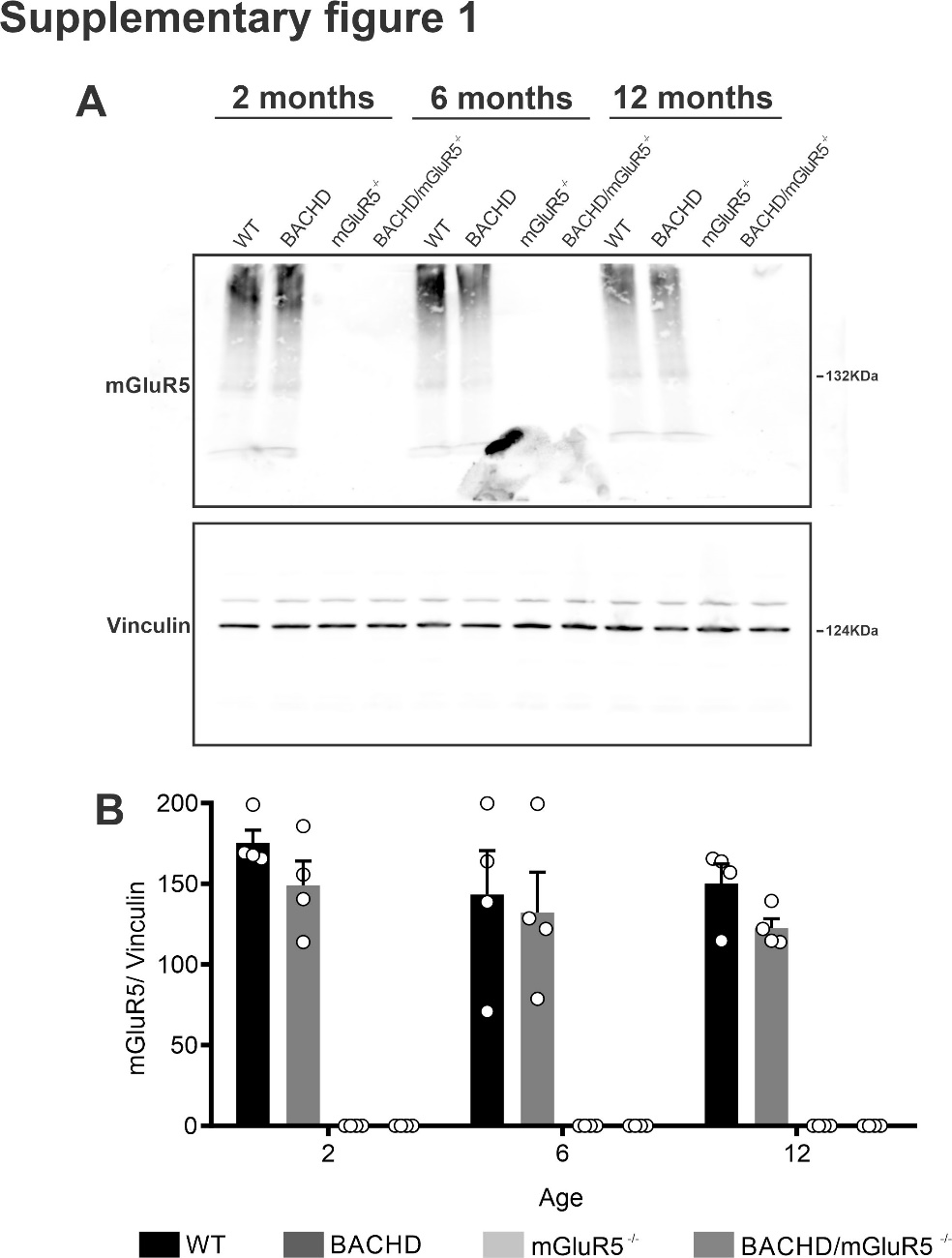


**Supplementary figure 1: Aging and mHTT expression do no alter the total mGluR5 expression in the hippocampus**. (A) Shown are representative immunoblots for mGluR5 (upper panel) and Vinculin (lower panel) expression in the hippocampus of wild-type (WT), BACHD, mGluR5^-/-^ and BACHD/mGluR5^-/-^ mice at 2, 6 and 12 months of age. 80 µg of total cell lysate was used for each sample. (B) Graph shows the densitometric analysis of mGluR5 normalized to vinculin expression in the hippocampus of WT, BACHD, mGluR5^-/-^ and BACHD/mGluR5^-/-^ mice at 2, 6 and 12 months of age (n=4). Data represent the means ± SEM.


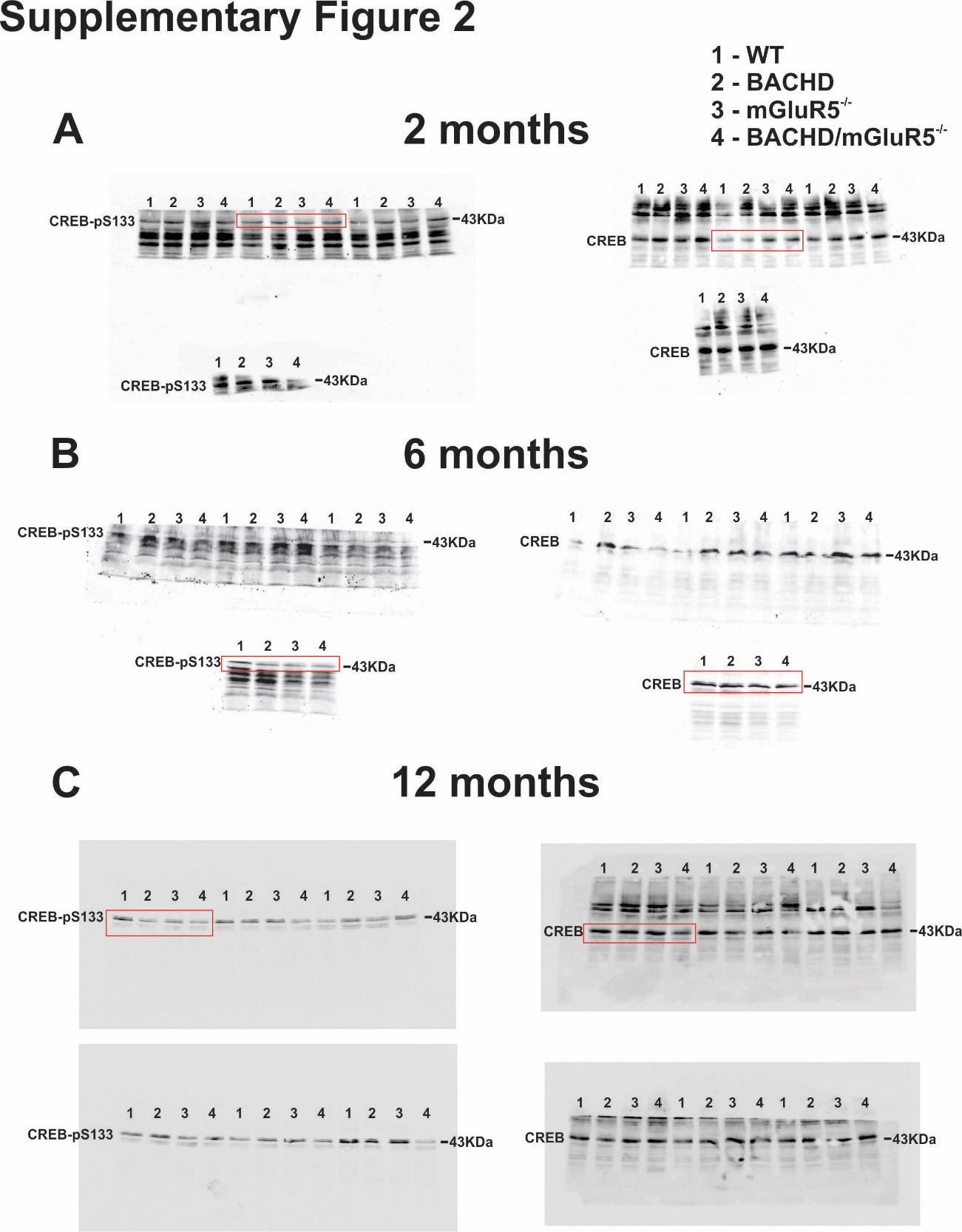


**Supplementary figure 2: Full-length western blots.** Shown are full-length immunoblots for CREB-p (S133) (left panels) and total-CREB (right panels) expression in the hippocampus of wild-type (WT) (1), BACHD (2), mGluR5^-/-^ (3) and BACHD/mGluR5^-/-^ (4) mice at 2 (A), 6 (B) and 12 (C) months of age. 80 µg of total cell lysate was used for each sample. Regions of the original blots used in main figures are denoted using red boxes. In the case of pCREB (A and B), the upper part of the membranes was covered before taking the images to avoid the interference of unspecific upper bands in the quantification of pCREB intensity.


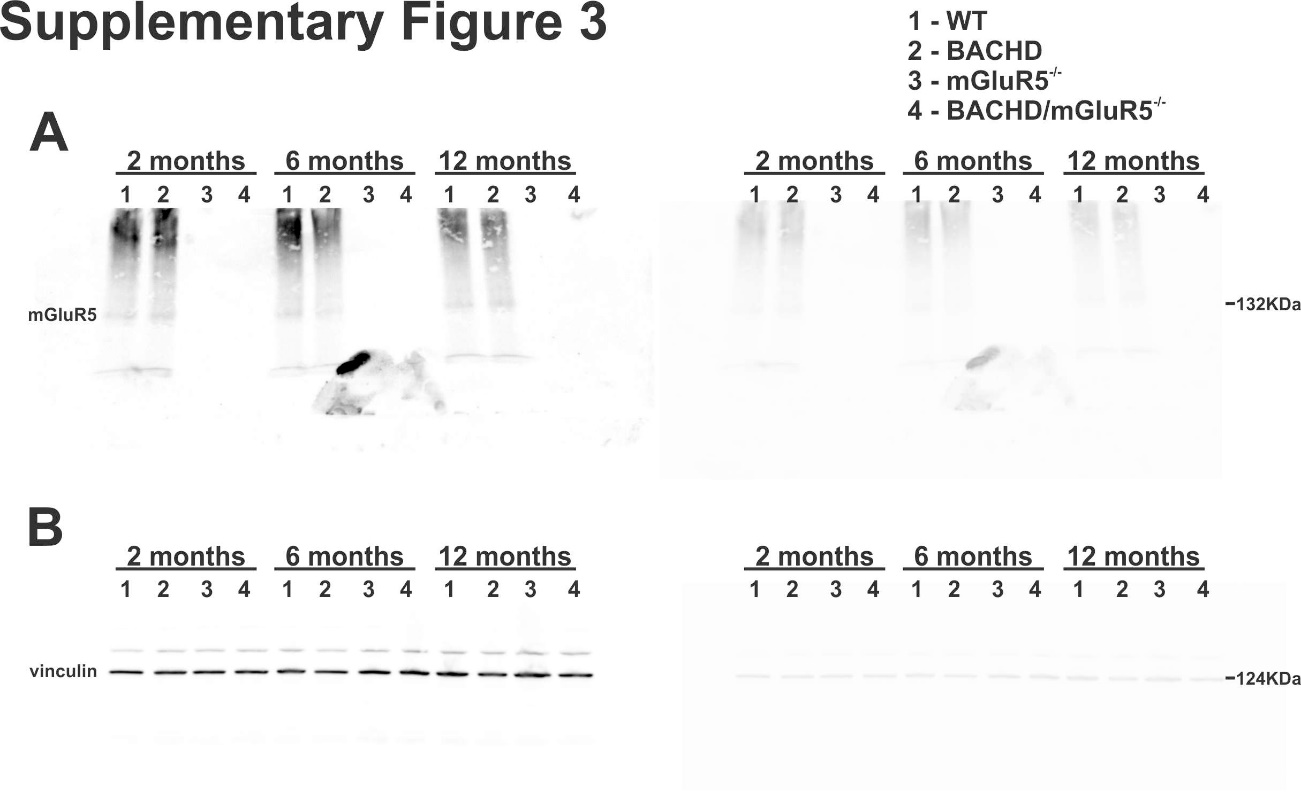


**Supplementary figure 3: Full-length western blots and multiple exposure images.** Shown are two additional images of mGluR5 (A) and vinculin (B) immunoblots, depicting different exposure levels of the same immunoblots shown on Supplementary Figure 1. 80 µg of total cell lysate was used for each sample from the hippocampus of wild-type (WT) (1), BACHD (2), mGluR5^-/-^ (3) and BACHD/mGluR5^-/-^ (4) mice at 2, 6 and 12 months of age.
